# Deubiquitinating Enzymes in Parkinson’s Disease

**DOI:** 10.3389/fphys.2020.00535

**Published:** 2020-06-03

**Authors:** Joy Chakraborty, Elena Ziviani

**Affiliations:** ^1^Department of Cell Biology and Physiology, CSIR-Indian Institute of Chemical Biology-TRUE Campus, Kolkata, India; ^2^Department of Biology, University of Padova, Padua, Italy

**Keywords:** Parkinson’s disease, ubiquitination, DUBs, mitophagy, neurodegeneration

## Abstract

Mitochondrial dysfunction and neurodegeneration have been directly correlated in many neurodegenerative disorders. Parkinson’s disease (PD) in particular has been extensively studied in this context because of its well-characterized association with mitophagy, a selective type of autophagy that degrades mitochondria. Mitophagy is triggered by ubiquitin modification of proteins residing on the surface of mitochondria. Therefore, mitophagy is subject to suppression by deubiquitination. In recent years, many deubiquitinase enzymes (DUBs) emerged as therapeutic targets to compensate hindered mitophagy in PD. It is reasonable that inhibition of specific DUBs should induce mitophagy by blocking deubiquitination of mitochondrial proteins, although the signaling pathway is not always that linear. The broad aspect suggests that there could be cross talks among DUBs, which may in turn have synergistic effect to rescue the disease progression. In this short review we have highlighted DUBs that hold therapeutic value in the field of neurodegenerative diseases, PD in particular.

## Parkinson’s Disease and Mitochondrial Quality Control: New Answers to an Old Riddle

The age-old question: how to address and slow down Parkinson’s disease (PD) progression, still remains elusive. The progressive decline in neuro- motor behaviors, which originates from specific loss of dopaminergic neurons in *Substantia Nigra Pars Compacta*, is the major concern. Dopaminegic neurons are known to be more vulnerable than other cell types because of their reliance on a specific subtype of Ca^2+^ channels to maintain their autonomous pacemaking activity (Chan et al., 2007), and their high-energy demand to sustain dopamine metabolism (Michel and Hefti, 1990; Zecca et al., 2003; Greenamyre and Hastings, 2004). Albeit fundamental for ATP synthesis to fuel cell needs (Nunnari and Suomalainen, 2012), oxidative phosphorylation can generate reactive oxidative species (ROS), and mitochondria can become source of cellular toxicity. The relationship between PD and mitochondria is further advocated by the fact that people with mutation in genes which are known to control mitochondrial degradation, like PINK1 and Parkin (Narendra et al., 2008; Geisler et al., 2010; Matsuda et al., 2010; Vives-Bauza et al., 2010; Bayne and Trempe, 2019; Biswas et al., 2020), develop a juvenile autosomal recessive form of PD (Kitada et al., 1998; Valente et al., 2004). The PINK1/Parkin pathway depicts that upon depolarisation of mitochondria, PINK1 recruits Parkin to mitochondria (Matsuda et al., 2010; Narendra D.P. et al., 2010; Vives-Bauza et al., 2010) to ubiquitinate specific proteins (VDAC, TOM20, MFN1, and MFN2, FIS1 among the most studied ones (Geisler et al., 2010; Tanaka et al., 2010; Ziviani et al., 2010; Chan et al., 2011; Yoshii et al., 2011; Sarraf et al., 2013; Junqueira et al., 2019). This event propels the organelle to the next step of mitophagy, if not pushed toward apoptosis (Ham et al., 2020). Ubiquitinated proteins on mitochondrial surface can directly interact with the autophagic isolation membrane via recruitment of autophagy receptors that contain conserved MAP1LC3/LC3-interacting regions (LIRs) (Johansen and Lamark, 2011). Several mitophagy receptors/adapters have been identified including p62/SQSTMA, FUNDC1, FKBP8, and BNIP3L/Nix, which are recruited to mitochondria under mitochondrial uncoupling stress conditions (Geisler et al., 2010; Narendra D. et al., 2010; Okatsu et al., 2010; Bhujabal et al., 2017), following hypoxia (Liu et al., 2012), or during activation of specific physiological pathways that control cell differentiation (Schweers et al., 2007; Sandoval et al., 2008; Novak et al., 2010). Not surprisingly, there are additional E3-ubiquitin ligases other than Parkin, which can ubiquitinate common targets on mitochondrial surface leading to Parkin-independent mitophagy. For example, MUL1 and Parkin share Mfn1 and two as common substrates, and they are both dependent on PINK1 for mitochondrial recruitment (Yun et al., 2014). Gp78 and Parkin also share common substrates, but whether Gp78 mitochondrial recruitment is PINK1 dependent or not is not fully clear (Fu et al., 2013). SIAH activation can trigger mitophagy independently of Parkin, but requires PINK1 to translocate to mitochondria (Szargel et al., 2016). Excavating these E3 ligases as alternatives to promote Parkin independent mitophagy is currently under intense investigation, and might hold therapeutic opportunities.

Many studies highlighted the importance of the ubiquitin proteasome system (UPS) for mitophagy execution (Yoshii et al., 2011). Proteosome-dependent degradation of mitochondrial pro-fusion proteins Mitofusins helps segregating dysfunctional mitochondrial from the mitochondrial network, and contributes to mitochondrial fission, which is required for efficient degradation (Tanaka et al., 2010). In addition, proteasome-dependent rapture of the outer mitochondrial membrane (Yoshii et al., 2011) exposes the inner mitochondrial membrane receptors of LC3, like Prohibitin two, (Wei et al., 2017) which prompt the formation of the autophagic isolation membrane.

It should be noted that there are other means by which mitochondrial quality control can be achieved that do not require the autophagy machinery. In particular, vesicles can bud from the mitochondrial network to deliver oxidized mitochondrial components to the lysosome for degradation (McLelland et al., 2014). These mitochondrial-derived vesicles (MDVs) represent a mechanism for selective removal of damaged mitochondrial parts, such as oxidized mitochondrial proteins, without degrading the entire organelle. An endosome-lysosome mitochondrial degradation pathway has also been recently described, in which damaged mitochondria are sequestered into early endosomes and delivered to lysosome for degradation (Hammerling et al., 2017). Both pathways are Parkin-dependent. Interestingly, canonical PINK1/Parkin pathway does not seem to be prevalent in neurons (Cai et al., 2012; Ashrafi et al., 2014; Lin et al., 2017). This raises the question whether neurons engage distinct mechanisms of mitochondrial quality control. Because neurons remain in non-dividing state, maintenance and recovery of mitochondrial integrity might be preferable to mitochondrial degradation (Puri et al., 2019).

In conclusion, different pathways are engaged to promote mitochondrial quality control. It is plausible that different cells deal with mitochondrial quality control differently, and the absence of one pathway and the level of compensation by the others may turn out to be the deciding factor for cell survival.

## DUBs and Mitochondrial Quality Control

**FIGURE 1:**
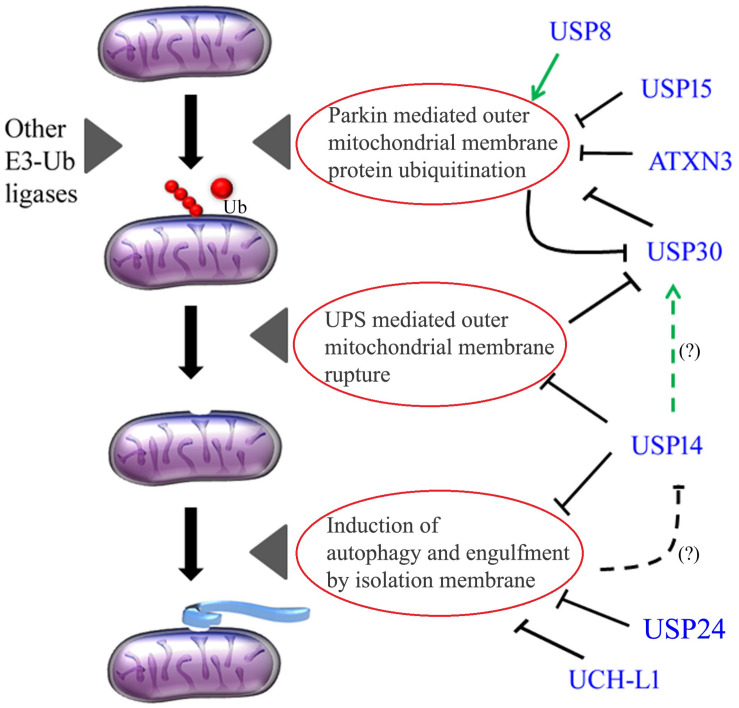
Deubiquinating enzymes in the regulation of mitophagy. Dotted lines with (?) indicate a potential functional interaction, which is yet to be demonstrated. USP8 positively regulates Parkin mitochondrial recruitment and mitophagy, whereas USP15, ATXN3, and USP30 negatively impact Parkin activity. Parkin has in turn a negative feedback loop on USP30. USP14 can tune the ubiquitin proteasome system (UPS) as well as autophagy. UCH-L1 and USP24 negatively influences autophagic membrane formation. It is yet to be demonstrated whether USP14 stability can be regulated by autophagy in a negative feedback loop (?), and whether USP14 can impact USP30 stability (?). Green arrows indicate positive impact while blunt lines indicate negative / antagonizing feedback.

Because ubiquitination plays an important role in mitochondrial quality control, the current field of therapeutic excavation targets those candidates, which can increase mitochondrial ubiquitination. To preserve mitochondrial ubiquitination long enough to activate mitophagy, one compound should either be able to enhance mitochondrial ubiquitination or inhibit deubiquitination. The class of enzymes, which counteracts ubiquitin ligases are known as deubiquitinating enzymes (DUBs). The general notion states that there are three different ways by which DUBs can affect mitophagy: (i) by regulating the stability of Parkin, (ii) by antagonizing the activity of Parkin and finally (iii) by regulating the level of proteasome activity and autophagy (Table 1 and Figure 1). Thus, these parameters were used as read-out for large-scale or DUBs-specific genetic screening to identify potential novel regulators of mitophagy. Durcan et al. used CCCP-induced Parkin translocation and a DUBs- specific RNAi-based approach, to identify USP8 as an important controller of stress-induced Parkin translocation (Durcan et al., 2014). USP8 interacts with the epidermal growth factor receptor, and regulates proliferation and differentiation. It also controls endosomal trafficking by ubiquitin-mediated sorting of the endocytosed cargoes (Mizuno et al., 2005; Row et al., 2006; Williams and Urbe, 2007). It has been previously demonstrated that auto-ubiquitination inhibits Parkin activity and translocation to depolarised mitochondria (Wauer and Komander, 2013; Kumar et al., 2015; Seirafi et al., 2015). USP8 deubiquitinates the K6 linked ubiquitin conjugates from Parkin, and contributes to the release of the auto-inhibited state of Parkin, to promote CCCP-induced Parkin translocation and Parkin-dependent mitophagy (Durcan et al., 2014). In a similar DUBs-specific RNAi-based cell screening, and using Mfn steady state levels as read out, USP8 and USP14 were identified to affect pathologically elevated Mfn protein levels. USP8 genetic and pharmacological inhibition improved mitochondrial function, climbing ability, life span, and dopaminergic neurons loss of two *Drosophila* models of mitochondrial dysfunction (von Stockum et al., 2019). Interestingly, USP8 knockdown also protects from α-synuclein–induced locomotor deficits and cell loss in an α-synuclein *Drosophila* model of PD (Alexopoulou et al., 2016), further supporting the potential therapeutic implication of USP8 inhibition. USP14 is among the DUBs, which remain in close association with the proteasome complex, and affect the degradation rate of proteasome-processed proteins. USP14 trims the ubiquitin chains one at a time, increasing the dwelling time of the protein on the proteasome (Lee et al., 2010). Thus, USP14 inhibition enhances proteasome activity that was found to impact autophagy, although its role in this context is controversial (Xu et al., 2016; Kim et al., 2018). Importantly, USP14 genetic or chemical inhibition exacerbates basal mitophagy in PINK1/Parkin independent manner, and proved to be protective in two well-established *Drosophila* models of PD (Chakraborty et al., 2018). Deubiquitinating enzyme Ataxin-3 (ATXN3), which mutation is associated to dominantly inherited ataxia (known as Machado–Joseph disease orMJD), was also found to directly interact with Parkin, and influence its ubiquitination. The mutant form of ATXN3 was reported to increase Parkin turnover by promoting its degradation via autophagy (Durcan and Fon, 2011; Durcan et al., 2011). ATXN3 regulates Parkin auto-ubiquitination by stabilizing the complex between Parkin and its E2 ubiquitin-conjugating enzyme, thus impeding the efficient transfer of ubiquitin from the E2 ubiquitin-conjugating enzyme to Parkin (Durcan et al., 2012). Increased Parkin turnover caused by mutant ATXN3 might explain some of the PD-related phenotypes that are observed in MJD, although a direct association between PD and ATXN3 mutation is yet to be well characterized. In another study that screened Flag-tagged human DUB cDNA library for CCCP-induced mitophagy, authors identified USP30 that efficiently antagonized mitochondrial loss (measured by immunostaining for TOM20). The study showed that Parkin and USP30 have common targets such as TOM20 and MIRO1, which are ubiquitinated by Parkin during CCCP-mediated mitophagy. Interestingly, USP30 is actually a Parkin substrate, and Parkin-dependent ubiquitination of USP30 facilitates proteasome-dependent degradation of the protein so that this DUB is kept at low levels when active Parkin is required to promote mitophagy. Knockdown of USP30 rescues defective mitophagy caused by parkin mutations and, more interestingly, improves mitochondrial phenotype of PINK1 and Parkin KO flies (Bingol et al., 2014). Thus, enhanced proteasome activity helps mitophagy via enhanced degradation of Parkin-opposing DUB USP30. Analysis of Parkin interactome by tandem affinity purification coupled to mass spectrometry identified DUB USP15 as physical Parkin interactor, but only in overexpressed conditions. The mechanism of action of USP15 is demonstrated to be by antagonizing Parkin ubiquitination on common mitochondrial targets, like Mfn2, and does not affect Parkin translocation or ubiquitination levels. Downregulation of USP15 promotes mitophagy in primary fibroblasts generated from skin biopsy of patients with PARK2 mutation, and rescues mitochondrial as well as locomotor phenotype of parkin RNAi flies (Cornelissen et al., 2014). Importantly, USP15 downregulation enhances basal mitophagy *in vivo* in parkin-downregulated flies measured by mt-keima, and specifically in neuronal cells. Because mitophagy increases with aging in wild type flies, but not in PINK1 or Parkin KO flies, the effect of USP15 KD on basal mitophagy has been interpreted as the mechanism of rescue seen in flies (Cornelissen et al., 2018).

**TABLE 1:** Deubiquitinase enzymes and Parkinson’s disease.

**Name**	**Key features**	**Link to PD**
USP8	Interacts with endosomal growth factor and regulates endodomal trafficking (Mizuno et al., 2005; Row et al., 2006).	Regulates Parkin activation (Durcan et al., 2014). Inhibition or downregulation protects against PD phenotypes in Drosophila model (Alexopoulou et al., 2016; von Stockum et al., 2019).
USP14	Inhibits proteasome and autophagy (Lee et al., 2010; Kim et al., 2018)	Inhibition leads to enhanced mitophagy and protect against PD phenotype in Drosophila models (Chakraborty et al., 2018).
Ataxin-3	Directly interacts with Parkin and influence the ubiquitination (Durcan and Fon, 2011; Durcan et al., 2011)	Might explain some of the PD related phenotypes in Machado–Joseph disease (Durcan et al., 2012).
USP30	Inhibits mitophagy by antagonizing Parkin (Bingol et al., 2014)	Knockdown protects against PD phenotype in *Drosophila* model (Bingol et al., 2014).
USP15	Inhibits mitophagy by antagonizing Parkin (Cornelissen et al., 2014).	Downregulation protects against PD phenotype in Parkin RNAi Drosophila model (Cornelissen et al., 2018).
UCH-L1	Regulates chaperon mediated autophagy and proteasome activity (Kabuta et al., 2008)	Mutant form of the protein protects against PD progression in MPTP induced mice model (Carmine Belin et al., 2007; Xilouri et al., 2012).
USP9X	Involved in cancer and Autoimmune disorders. Reversely correlated to mon-ubiquitinated α-Synuclein levels (Rott et al., 2011)	Yet to be identified.
USP24	Negative regulator of autophagy (Thayer et al., 2020)	Regulates neurite growth in dopaminergic cells (Li et al., 2006).

Among the regulators of autophagy and proteasome, another DUB UCH-L1 was found to be associated with PD. UCH-L1 acts on polyubiquitin chain, and increase availability of free monomeric ubiquitin to the ubiquitin proteasome system, thus increasing proteasome-dependent degradation. Interestingly, the I94M mutation in UCH-L1 has been found in autosomal dominant PD patients (Leroy et al., 1998; Kabuta et al., 2008), and reduced mRNA and protein levels of this DUB were found in PD post-mortem samples from frontal cortex and medulla oblongata. Because UCH-L1 influences the activity of the proteasome, and it is associated with pathological α-Synuclein accumulation, these results led to the conclusion that UCHL-1 contributes to abnormal protein aggregation (Barrachina et al., 2006). In perfect agreement with this, it was reported that UCH-L1 directly interacts with chaperone-mediated autophagy (CMA), by physically binding to LAMP-2A, Hsp70 and Hsp90 (Kabuta et al., 2008). It should be noted that a protective S18Y UCH-L1 variant has also reported to be reversely correlated to the disease onset (Carmine Belin et al., 2007), although contrasting works on this topic have been published (Healy et al., 2006). The protective feature of the mutation has been specifically studied in the dopaminergic system of MPTP induced mouse model of PD, and is attributed to its antioxidant property (Xilouri et al., 2012). Several lines of evidence indicate that DUB USP9X also plays a role in the etiology of PD, and other neurodegenerative diseases. USP9X is an eclectic DUB that is highly conserved from *Drosophila* to mammals. It plays a role in human cancer, developmental and autoimmune diseases. In the context of neurodegeneraiton, USP9X has been shown to direct the autophagic degradation of α-Synuclein by deubiquitinating its monoubiquitinated form (Rott et al., 2011). Interestingly, in brain tissue, USP9X colocalises with α-Synuclein inclusions, and PD patients present reduced levels of USP9X. It is believed that decreased levels of USP9X result in accumulation of monoubiquitinated α-Synuclein, which are more prone to aggregate and caused neuronal toxicity. Finally, DUB USP24 was recently identified as a negative regulator of autophagy by affecting steady state levels of autophagy factor ULK1. USP24 downregulation promotes ULK1 stability, increases the autophagic flux and prevents age-dependent neurite growth decline of an iPSCs-derived dopaminergic neuronal model (Thayer et al., 2020). Interestingly, USP24 genetic polymorphisms is correlated to PD onset (Li et al., 2006; Haugarvoll et al., 2009).

The potential involment of these DUBs in mitohondrial autophagy has not been yet investigated.

## Conclusion

The mammalian genome encodes for more than one hundred DUBs, indicative of large substrate specificity. The scenario depicts that there might be many overlapping pathways, which are controlled by multiple DUBs and may impact on PD development or progression. The cross talk between USP8, USP15, and ATXN3 can coordinate the activity of these DUBs, and ultimately control Parkin stability and activation (Figure 1). Interestingly, Parkin and USP30 have negative feedback loops on each other and might be very finely tuned under physiological conditions (Figure 1). As USP30 is a substrate of the proteasome, USP14 has the potential of controlling Parkin stability and Parkin-dependent mitophagy. Simultaneous studies to investigate the level and activity of these DUBs in PD models will be instrumental to further delineate the compensatory effects that might be crucial for the disease progression.

## Author Contributions

JC and EZ wrote the review.

## Conflict of Interest

The authors declare that the research was conducted in the absence of any commercial or financial relationships that could be construed as a potential conflict of interest.

## Abbreviations

ATXN3: Ataxin-3
BNIP3L: BCL2/adenovirus E1B 19 kDa protein-interacting protein 3-like
CCCP: carbonyl cyanide m-chlorophenyl hydrazone
CMA: chaperone-mediated autophagy
DUBs: deubiquitinase enzymes
FIS1: fission 1
FKBP8: FK506-binding protein 8
FUNDC1: FUN14 domain-containing protein 1
Gp78: glycoprotein 78
Hsp: heat shock protein
iPSC: induced pluripotent stem cell
LAMP2: lysosome-associated membrane glycoprotein 2
LIR: LC3 interacting region
Map1lc3b: microtubule-associated proteins 1A/1B light chain 3B
MDV: mitochondrial-derived vesicle
MFN: Mitofusin
MIRO1: mitochondrial Rho GTPase 1
MJD: Machado–Joseph disease
MPTP: 1-methyl-4-phenyl-1,2,3,6-tetrahydropyridine
MUL1: mitochondrial E3 ubiquitin protein ligase 1
Nix: NIP3-like protein X
PARK2: Parkinson disease protein 2
PD: Parkinson’s disease
PINK1: PTEN-induced kinase 1
ROS: reactive oxidative species
SIAH: seven in absentia homolog
SQSTM1: P62/sequestosome-1
TOM: translocase of outer membrane
UCH-L1: ubiquitin carboxyl-terminal hydrolase isozyme L1
UPS: ubiquitin proteasome system
USP: ubiquitin-specific-processing protease
VDAC: voltage-dependent anion channel.
